# Non-coding RNAs and retroviruses

**DOI:** 10.1186/s12977-018-0403-8

**Published:** 2018-02-09

**Authors:** Xu Zhang, Xiancai Ma, Shuliang Jing, Hui Zhang, Yijun Zhang

**Affiliations:** 1 Institute of Human Virology, Zhongshan School of Medicine, Sun Yat-Sen University, Guangzhou, 510080 China; 2 Key Laboratory of Tropical Disease Control of Ministry of Education, Zhongshan School of Medicine, Sun Yat-Sen University, Guangzhou, 510080 China; 3 Guangdong Engineering Research Center for Antimicrobial Agent and Immunotechnology, Zhongshan School of Medicine, Sun Yat-Sen University, Guangzhou, 510080 China; 4 Section of Infectious Diseases, Department of Internal Medicine, Yale University School of Medicine, New Haven, CT 06520 USA

**Keywords:** Non-coding RNA, Retroviruses, Viral life cycle, Virus latency, MicroRNA, Long non-coding RNA

## Abstract

Retroviruses can cause severe diseases such as cancer and acquired immunodeficiency syndrome. A unique feature in the life cycle of retroviruses is that their RNA genome is reverse transcribed into double-stranded DNA, which then integrates into the host genome to exploit the host machinery for their benefits. The metazoan genome encodes numerous non-coding RNAs (ncRNA), which act as key regulators in essential cellular processes such as antiviral response. The development of next-generation sequencing technology has greatly accelerated the detection of ncRNAs from viruses and their hosts. ncRNAs have been shown to play important roles in the retroviral life cycle and virus–host interactions. Here, we review recent advances in ncRNA studies with special focus on those have changed our understanding of retroviruses or provided novel strategies to treat retrovirus-related diseases. Many ncRNAs such as microRNAs (miRNAs) and long non-coding RNAs (lncRNAs) are involved in the late phase of the retroviral life cycle. However, their roles in the early phase of viral replication merit further investigations.

## Background

### The classification and life cycle of retroviruses

Retroviruses represent a large and diverse family of enveloped RNA viruses defined by common taxonomic denominators that include structure, composition, and replicative properties [1]. A key feature of the retroviral life cycle is that the RNA genome is reverse-transcribed to double-stranded DNA, which is subsequently integrated into the host genome and turns to a provirus. The viral genes are transcribed from the integrated proviral DNA to produce proteins and genomic RNA required to assemble the progeny viral particles. Retroviruses are further subdivided into seven groups (genus) defined by their evolutionary relatedness [2]. Retroviruses in five of these groups have oncogenic potential (formerly referred to as oncoviruses), and the other two groups are lentiviruses and spumaviruses. The representative of the lentivirus family is the human immunodeficiency virus type 1 (HIV-1), the causative agent of acquired immunodeficiency syndrome (AIDS). There are over 36 million people living with HIV-1 worldwide, with approximately 2.1 million new infections being reported in 2015. To date, there is no cure for AIDS because of the existence of the HIV reservoir. The latent reservoir is a group of HIV-infected cells (mainly resting CD4^+^ T cells) that do not actively produce new HIV-1, but could produce virus again upon stimulation [3].

**Fig. 1:**
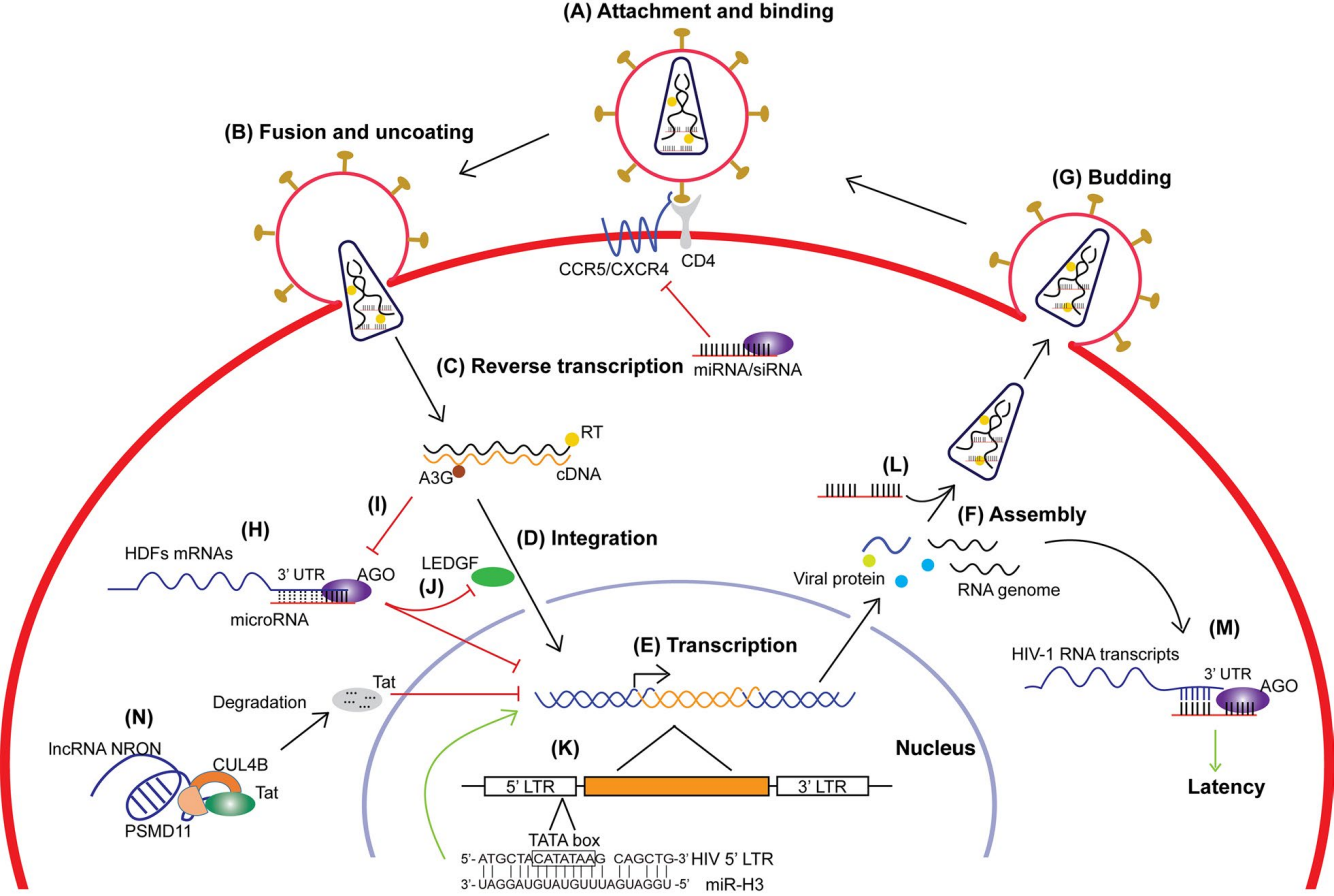
Non-coding RNAs and HIV-1 life cycle. The life cycle of HIV-1 includes: attachment and binding, fusion and uncoating, reverse transcription, integration, transcription, assembly, budding and latency. The roles of representative microRNAs and long non-coding RNAs in HIV-1 life cycle are shown. *RT* reverse transcriptase; *A3G* APOBEC3G; *AGO* Argonaute protein; *LEDGF* lens epithelium-derived growth factor or p75; *HDFs* host dependency factors; *miRNA* microRNA; *siRNA* small interfering RNA

The life cycle of retroviruses can be simply divided into the early and late phases. The early phase refers to the steps from cell binding to integration of the viral cDNA in the host genome, whereas the late phase begins with the expression of viral genes and is followed by the assembly, release, and maturation of progeny virions [4]. For HIV-1, the lifecycle can be briefly divided into seven steps: (1) attachment and binding, (2) fusion and uncoating, (3) reverse transcription, (4) integration, (5) transcription, (6) assembly, and (7) budding (Fig. 1A–G). Steps 1–4 represent the early phase, and steps 5–7 represent the late phase of a typical retrovirus life cycle. In addition, there is a special state of the retrovirus life cycle called latent infection. Under such conditions, the proviral DNA is transcriptionally inactive without producing infectious viral particles. As the host CD4^+^ T cells are activated, the phase of latent HIV infection can be reversed to productive infection [3, 5]. Numerous host factors are involved in the life cycle of retroviruses, being either indispensable or restrictive [6].

Virology and RNA biology have reciprocally influenced each other for decades [7]. The capping of eukaryotic mRNA was first discovered in reovirus and vaccinia virus [8]. Splicing and then alternative splicing were first demonstrated by analysis of adenoviral transcription [9, 10]. Analysis of viral systems, in particular picornaviruses, led to the first description of an internal ribosome entry site (IRES) [7]. Retroviruses have also played an important role in understanding the export of mRNAs from the nucleus to the cytoplasm. For host transcripts, only fully spliced mRNAs can be exported to the cytoplasm. However, for some retroviruses such as HIV-1, both spliced and unspliced transcripts need to be exported to the cytoplasm to produce viral proteins or serve as genomic RNA. HIV-1 encodes a special protein called Rev that exports its unspliced mRNA transcripts containing the Rev Response Element (RRE) to the cytoplasm [11]. To accomplish this function, Rev harbors a nuclear export signal (NES), which was the first NES described, and still remains the prototype of the most common class of NESs [12]. Compatible with such significant progress in virus research, the recent advances in non-coding RNA (ncRNA) research have greatly extended our understanding of viruses.

### The classification and functions of non-coding RNAs

Genomic studies have demonstrated that only two percent of the human genome codes for proteins, whereas the majority codes for numerous non-protein coding RNAs (or non-coding RNA, ncRNA) [13]. Based on their function, non-coding RNAs can be divided into two major groups: (1) housekeeping non-coding RNA such as ribosomal RNA (rRNA), transfer RNA (tRNA), small nuclear RNA (snRNA), and small nucleolar RNA (snoRNA); and (2) regulatory non-coding RNA such as microRNA (miRNA), long non-coding RNA (lncRNA), and piwi-interacting RNA (piRNA) [14]. The housekeeping non-coding RNAs are mainly involved in the basic biological processes in cells, such as snRNA for pre-mRNA splicing, rRNA and tRNA for mRNA translation, snoRNA for rRNA methylation/pseudouridylation. However, the regulatory non-coding RNAs exert more diverse and sophisticated functions such as miRNA for regulating gene transcription and translation, lncRNA for modifying epigenetic signatures of chromatin, and piRNA for silencing transposons [14]. Unlike housekeeping ncRNAs, the expression of regulatory ncRNAs is usually tissue-specific and their regulation is gene-specific, which is mostly enabled by sequence-specific interactions between the ncRNA and its target(s) [15]. During viral infection, the regulatory ncRNAs exhibit more profound changes in expression and sophisticated functions compared to the housekeeping ncRNAs [16].

### RNA interference and microRNA

RNA interference (RNAi) was proposed to initially evolve in plants and invertebrates as a native immune response against viruses [17]. In plant and invertebrate cells, the infection of all RNA viruses, except retroviruses, leads to the generation of perfectly base-paired long double stranded RNAs (dsRNA), which are cleaved by the exonuclease Dicer into ~ 22 bp short dsRNAs named small interfering RNA (siRNA) [17]. This is also true for some DNA viruses. One strand of the siRNA duplex is loaded into the RNA-induced silencing complex (RISC), where it guides RISC to mRNA containing complementary sequence [18]. Since these virus-derived siRNAs are fully complementary to viral mRNAs, the RISC binding guided by these siRNAs will silence their cognate viral RNAs and inhibit virus replication. In plants and nematodes, this antiviral response can be further amplified by generating a secondary wave of siRNAs through a mechanism involving RNA-dependent RNA polymerases (RdRPs) [19, 20]. In mammalian cells, RNAi mediated by siRNA is no longer a major form of the antiviral response. Instead, interferon (IFN) response and it-induced systematic antiviral state play a key role (reviewed in Ref. [21]). However, the RNAi mechanism was retained to generate endogenous microRNA (miRNA) and regulate the expression of a significant amount of genes. MicroRNAs represent an important class of regulators in many crucial biological processes in animals, plants, fungi, and viruses [22, 23]. In the nucleus, primary miRNAs (pri-miRNAs) are transcribed and processed by Drosha and its cofactor DGCR8 (also known as Pasha, located in the DiGeorge syndrome chromosomal region) to generate precursor miRNAs (pre-miRNAs) [24], which are then exported to the cytoplasm [25] and further sliced by Dicer to generate ~ 22 nt mature miRNAs [26]. Like siRNAs, one strand of mature miRNAs is bound by Argonaute (AGO) proteins and loaded into the RISC [27] for guiding the RISC to the 3′ UTR of mRNAs to suppress translation or induce degradation. Unlike siRNAs, which fully complement to their target mRNA, most miRNAs bind to the target mRNA mainly through the “seed sequence”, residues 2–8 of the guide miRNAs (Fig. 1H). The regulatory target sequences of miRNA have been extended to the 5′ UTR [28] and the coding region [29] of mRNAs to suppress translation. Moreover, miRNAs could switch from suppressor to activator of translation by targeting the 5′ UTR [30] or 3′ UTR [31] of certain mRNAs. In addition to regulating mRNA translation in the cytoplasm, mounting evidence reveals that the small RNA-Ago pathway can also positively regulate gene expression by targeting gene promoters in the nucleus, a phenomenon termed as RNA activation (RNAa), which is evolutionarily conserved from *Caenorhabditis elegans* to human [32–35].

### Long non-coding RNAs

The ENCODE (The Encyclopedia of DNA Elements) Project led to the discovery of a large proportion of long transcripts from the human genome that do not code proteins (long non-coding RNAs, lncRNAs) [36]. lncRNAs can be operationally defined as RNA transcripts larger than 200 bp without any coding potential [37]. They appear in the genome in three major forms: antisense lncRNAs, intronic lncRNAs, and intergenic lncRNAs (also termed large intervening noncoding RNAs or lincRNAs). One of the major functions of lncRNA is to regulate gene activity through interactions with chromatin, especially to suppress gene expression. For instance, gene expression on the X-chromosome can be inactivated by the lncRNA Xsit [38]. Although the mechanisms of lncRNA-mediated gene regulation are diverse, three main mechanistic themes have been drawn from dozens of examples (see review by Rinn and Chang [37]). These include decoys, scaffolds, and guides. Notably, when an lncRNA guide protein complex to the target site on chromatin, how does the lncRNA recognize and interact with the target DNA is still unclear. In addition to using DNA-binding protein(s) as adapter, the RNA–DNA direct interaction through RNA:DNA hybrid or RNA:DNA:DNA triplex are also possible forms requiring further investigation [39]. Increasing attention has been given to lncRNAs due to their strong connection with the development of various diseases, especially cancers. The elevated expression of the lncRNA Hox transcript antisense intergenic RNA (HOTAIR) has been observed in human breast, colon, and liver cancers, and facilitates cancer metastasis in vivo [40–42]. Extensive studies on the role of lncRNA in cancers may provide useful insights for the study of lncRNAs in the retrovirus system.

In this review, we discuss recent advances in the study of non-coding RNAs that play important roles in the life cycle of retroviruses, with special focus on miRNAs and lncRNAs (Tables 1, 2). Moreover, the treatment of retrovirus-related diseases can also benefit from the study of non-coding RNAs.

**Table 1:** Retroviral and host microRNA

	Virus	Name	Viral target(s)	Cellular target(s)	Function	References
Viral microRNA	HIV-1	miR-H3	HIV-1 5′ LTR TATA box		Enhance HIV-1 5′ LTR transcription	[77]
		TAR-derived miRNA		ERCC1, IER3, Caspase 8, Aiolos, Ikaros, Nucleophosmin (NPM)/B23	Protect the infected cells from apoptosis, maintain balance between apoptosis and cell survival	[70, 71, 75, 76]
		miR-N367	Nef		Block HIV-1 Nef expression	[72]
		vmiRNA#1-5		Predicted	Unknown	[68]
	BLV	B1-5		Bovine HBP1, PXDN	Mimic host miR-29, tumorigenesis	[66]
	BFV	miR-BF2-5p miR-BF1-5p miR-BF1-3p			Unknown	[85]
	ALV-J	E (XSR) miRNA			Unknown, possible roles in myeloid leukosis associated with ALV-J	[86]
Host microRNA		miR-29a miR-29b miR-149 miR-378 miR-324-5p	HIV-1 Nef, Vpr, Env, Vif transcripts		Downregulate the expression of Nef protein and interfere with HIV-1 replication	[119–121]
		miR-326	Nef ORF located in the 3′ U3 of HIV-1 transcripts		Moderate HIV-1 replication in human cells	[122]
		miR-423 miR-301a miR-155	HIV-1 genome		Repress viral gene expression	[123]
		miR-28 miR-125b miR-150 miR-223 miR-382	HIV-1 mRNA 3′ ends		Contribute to HIV-1 latency	[46]
		miR-146		CXCR4	Inhibit HIV-1 infection	[47, 48]
		miR-155		ADAM10, TNPO3, Nup153, LEDGF/p75	Inhibit HIV-1 infection	[60]
		miR-198		Cyclin T1 mRNA	Restrict HIV-1 replication in monocytes	[102]
		Let-7i		IL-2 promoter TATA box	Activate IL-2 transcription, involved in HIV-1 infection-induced CD4^+^ T cell depletion	[78, 137]

**Table 2:** Retroviral and host LncRNA

	Virus	Name	Viral targets	Cellular targets	Function	References
Viral lncRNA	HIV-1	HIV-1-encoded antisense RNA	HIV-1 5′ LTR		Alter the epigenetic landscape and silence the HIV-1 promoter	[84]
	HERV-H	HERVH RNA		p300, OCT4	Activate neighboring genes and in turn regulating pluripotency-related genes	[93]
	HTLV-1	HBZ		E2F1	Promote T cell proliferation	[97, 98]
Host lncRNA		NRON	Tat	NFAT	Induce the degradation of Tat and contribute to HIV-1 latency; suppress NFAT-mediated viral gene activation	[132, 135]
		NEAT1	HIV-1 unspliced RNAs	p54nrb, PSF, Matrin3, RBM14	Inhibit nucleus-to-cytoplasm export of Rev-dependent instability element (INS)-containing HIV-1 mRNAs	[104, 106, 108–110]
		7SK snRNA		P-TEFb	Negatively regulate HIV-1 Tat transcriptional activation by specifically sequestering P-TEFb	[111, 112]
		7SL RNA	HIV-1 Gag	APOBEC3G	Enable efficient packaging of APOBEC3G into virions	[128]

## Non-coding RNAs and the early phase of the retrovirus life cycle

The early phase of retrovirus life cycle includes binding and fusion of virions to host cells, reverse transcription of viral genomic RNA into cDNA, and its integration in the host chromosome (Fig. 1A–D). During the binding and fusion steps, viral Env proteins play a leading role. The HIV-1 Env trimer first binds to the cell surface CD4 receptor and induces conformational changes that allows binding to the CCR5 or CXCR4 co-receptor, which mediates the fusion of viral and cell membranes. The study on how non-coding RNAs are involved in these steps is missing. In general, RNA is quite unstable in the extracellular environment and ready to be degraded by RNase. However, several reports showed that some ncRNAs, e.g. miRNAs or lncRNAs, are circulating in the blood, which can serve as biomarkers of various diseases [43, 44]. The improved stability of these ncRNAs could be due to their small size or special secondary structures [37]. An implication from the existence of these extracellular RNAs is that the non-coding RNAs can be involved in the early steps of retroviral infection, such as the binding and fusion of virions to the host cells. A possibility is that these extracellular ncRNAs could enter the target cells along with the viruses and affect viral infectivity. The virion-associated ncRNAs might also play certain roles in these steps. A small subset of host miRNAs are shown to be concentrated in the HIV-1 virions by up to 115 folds [45]. Notably, three of the packaged miRNAs: miRNA-382, miRNA-223, and miRNA-150, were able to interact with the 3′ UTR of HIV-1 mRNA as well as contribute to HIV-1 latency [46] (see discussion in the non-coding RNAs and retrovirus assembly section below). However, their functions within the virions remain to be determined. It is interesting to investigate whether the non-coding RNAs packaged in virions are involved in viral fusion and uncoating steps, considering they have been protected by the viral particle from the damage caused by the extracellular environment. As mentioned above, these virion-enriched ncRNAs could also affect the expression of entry- or uncoating-related proteins.

As the co-receptor is important for HIV-1 entrance, the dysregulation of CCR5 or CXCR4 significantly affects HIV-1 infection. It has been shown that CXCR4 is a direct target of miR-146a in human hematopoietic normal and leukemic cells; and the transcription factor PLZF (promyelocytic leukemia zinc finger) has been identified as a repressor of miR-146a expression in megakaryocytic (Mk) cells [47]. These authors further reported that the miR-146a upregulation by AMD3100 treatment or PLZF silencing decreases CXCR4 protein expression and prevents HIV-1 infection in monocytic cell line and CD4^+^ T lymphocytes [48]. However, the suppression of CXCR4 by miR-146 was not confirmed at least in MT2 cells [49]. At present, there is no solid evidence for CCR5 to be regulated by any cellular or viral microRNA and the HIV-1 infection is therefore affected.

For HIV-1, the initiation of reverse transcription is coupled with the uncoating of the viral core [50]. It is well known that tRNA_Lys3_ serves as the primer for reverse transcription of viral genomic RNA. In addition to primer tRNA, the reverse transcription complex (RTC) contains multiple proteins, including viral proteins MA (matrix protein, p17), CA (capsid protein, p24), NC (nucleocapsid protein, p7), IN (integrase), and Vpr [51]. The mature CA protein most likely provides the overall structure of the RTC [52, 53]. Only a few host proteins have been identified from the RTC. The cellular protein cyclophilin A (CypA) was shown to play a critical role in the correct disassembly of the HIV-1 core early after infection [54]. The APOBEC3 family is involved in the restriction of retrovirus reverse-transcription, functioning as cytosine deaminases that introduce hyper G to A mutations to the sense strand of proviral cDNA [55]. While no report shows the expression of these host factors is directly regulated by miRNA or other ncRNAs, our studies have shown that APOBEC3G and its family members cause derepression of miRNA-mediated protein translation inhibition [56], which is through interfering with the interaction between Argonaute-2 and MOV10 [57] (Fig. 1I). These findings imply that there could be a complex regulation network involving retroviruses, APOBEC3 proteins and the host miRNA pathway. It will be interesting to investigate the roles of lncRNAs during the assembly of the RTC, e.g. as a scaffold for the complex assembly. It is also intriguing to explore the roles of virion-associated miRNAs during reverse transcription, given that these miRNAs could potentially bind to the 3′ UTR of viral genomic RNA and be packaged into the virions.

At the late stage of the reverse transcription, the RTC transitions into a pre-integration complex (PIC), which is subsequently transported into the nucleus. Similarly, the integration of viral DNA into the host chromosome involves many host factors, such as proteins that help the transfer of viral DNA to nucleus and the integration of viral DNA into the host chromosome. For example, the cellular transcriptional coactivator lens epithelium-derived growth factor (LEDGF)/p75 is an essential HIV integration cofactor, which forms stable tetramers and associates with HIV-1 integrase [58, 59]. The stimulation of polyinosinic-polycytidylic acid [poly (I:C)] and bacterial lipopolysaccharide (LPS), the ligands for toll-like receptor 3 (TLR3) and TLR4, respectively, are known to decrease HIV-1 infection in monocyte-derived macrophages (MDMs), but the mechanism was unclear. It has been shown that stimulation with poly (I:C) upon TLR3 in MDMs leads to the upregulation of miR-155 expression, and consequently the downregulation of its target proteins including ADAM10, TNPO3, Nup153, and LEDGF/p75. This study indicates that a TLR3-induced miRNA exerts an anti-HIV-1 effect by targeting several HIV-1 dependency factors to inhibit HIV-1 infection [60] (Fig. 1J).

Compared to the late phase, less is known about the early steps of retrovirus life cycle, as reviewed by Nisole and Saib [4]. Accordingly, the roles of non-coding RNAs played during the early phase of retrovirus life cycle are still largely unknown.

## Non-coding RNAs and the late phase of the retrovirus life cycle

### Retrovirus-encoded non-coding RNAs

After integration, the provirus starts to transcribe viral genes. The transcription of the proviral genome leads to generation of diverse products including genomic RNA, spliced mRNAs, structural proteins, accessory proteins, and some non-coding RNAs [61]. These viral parts play various roles in the late stage of retroviral life cycle, and some of them are essential parts of the progeny viruses. Below we describe the roles played by virus-encoded and cellular non-coding RNAs in the late stage of the retroviral life cycle.

#### HIV-1-encoded non-coding RNAs

Virus-encoded miRNAs were initially identified from Epstein–Barr viruses (EBV) [62]. Since then, an increasing number of virus-encoded miRNAs have been identified [17, 63]. Most of these miRNAs were reported in DNA viruses such as Herpes and Polyoma viruses, but rarely in RNA viruses [17]. Because of the rapid developments of next-generation sequencing (NGS, or deep-sequencing) technology, which is much more sensitive and quantitative than the conventional cDNA clone sequencing method, more RNA virus-derived miRNAs have been discovered, especially from HIV-1, West Nile Virus (WNV), and Bovine Leukemia Virus (BLV) [64–67].

It has been reported that HIV-1 encodes miRNAs and other small RNAs. Bennasser et al. first performed a computational prediction of HIV-1-encoded miRNAs and found five pre-miRNA candidates [68]. Subsequently, several groups identified miRNAs from the HIV-1 negative regulatory factor (Nef) and trans-activation response (TAR) element [69–72]. Through the NGS method, a number of HIV-1-encoded small RNAs were discovered, some of which exhibit features of miRNA siRNA [73–75]. HIV-1-derived small RNAs have been shown to modulate cellular and/or viral gene expression. The TAR-derived miRNA protects the infected cells from apoptosis by downregulating cellular genes involved in apoptosis [71, 76]. A Nef-derived miRNA-miR-N367 blocks HIV-1 Nef expression in vitro [72].

The low abundance of HIV-1-derived small RNAs has fueled the debate about the existence and function of HIV-1-encoded miRNAs [17]. A possible explanation of the low abundance of HIV-1-derived miRNAs is that the virus has been evolving to escape from or counteract the RNAi-mediated immune surveillance. HIV-1 has developed special ways to manipulate the host RNAi, such as using a non-processed transcript to produce miRNAs and targeting viral promoter DNA for the regulation of gene expression. Harwig et al. revealed that non-processive transcription from the HIV-1 LTR promoter results in the production of TAR-encoded miRNA-like small RNA [75]. Dicer cleaves these TAR RNAs and the viral transactivating regulatory protein (Tat) stimulates this processing. Through this special biogenesis pathway, HIV-1 produces the TAR-derived miRNA without cleavage of its RNA genome. Through a strategy combining in silico prediction and NGS, our group identified a novel HIV-1-encoded miRNA, miR-H3 [77]. miR-H3 is located in the mRNA region encoding the active center of reverse transcriptase (RT), and exhibits high sequence conservation among HIV-1 subtypes. The overexpression of miR-H3 increases viral production, while mutations in the miR-H3 sequence significantly impair the replication of HIV-1_NL43_, suggesting that it is a viral replication-enhancing miRNA. Interestingly, miR-H3 targets the HIV-1 5′ LTR TATA box and sequence-specifically activates the viral promoter transcription. This is the first report of the promoter TATA box-targeting miRNAs that activate gene transcription [77]. These miRNAs might directly interact with the TATA box in the RNA Polymerase II pre-initiation complexes (PICs) and facilitate transcription initiation [78]. In contrast to cytoplasmic miRNAs that need a quite high expression level to bind and regulate their massive mRNA targets, the abundance of the TATA box-targeting miRNAs required for regulating promoter activity is significantly reduced, given that the copy number of their targets in the nucleus is very limited (Fig. 1K).

The secondary structure of the entire HIV-1 RNA genome is quite complicated [79]. Several groups recently found that HIV-1 transcribes not only full-length genomic RNA and multiple spliced mRNAs, but also some antisense RNAs [65, 80–82]. Some of the HIV-1-encoded antisense RNAs can suppress HIV-1 expression [83]. Particularly, one of these suppressive HIV-1-encoded antisense RNAs is a lncRNA [84]. Further experiments showed that this lncRNA could alter the epigenetic landscape of the HIV-1 promoter by recruiting DNA methyltransferase 3A (DNMT3A), enhancer of zeste homolog 2 (EZH2), histone deacetylase 1 (HDAC1), and euchromatic histone-lysine N-methyltransferase 2 (EHMT2, also known as G9a) to the HIV-1 5′ LTR. These proteins recruited by the antisense lncRNA lead to an epigenetically silenced state of the viral promoter, which is characterized by multiple suppressive epigenetic markers including histone deacetylation, H3K9 dimethylation, and H3K27 trimethylation [84].

#### Other retrovirus-derived non-coding RNAs

Interestingly, to prevent the cleavage of their RNA genome, some retroviruses use an alternative RNA source as miRNA precursor. A deltaretrovirus bovine leukemia virus (BLV) uses RNA Pol III transcripts to produce five miRNAs in a Drosha-independent manner [66]. Whisnant et al. identified three miRNAs from bovine foamy virus (BFV), a member of the spumavirus subfamily of retroviruses, in both BFV-infected cultured cells and BFV-infected cattle. All three viral miRNAs are generated from an ~ 122-nucleotide (nt) pri-miRNA encoded within the BFV long terminal repeat U3 region. This BFV pri-miRNA is also transcribed by RNA polymerase III [85]. In the avian leukosis virus subgroup J (ALV-J), an alpharetrovirus, a miRNA encoded by the exogenous virus-specific (E or XSR) region has been described. Unlike the above reported miRNAs, this miRNA is generated by the canonical miRNA biogenesis pathway. The pri-miRNA is transcribed by RNA Pol II and requires Drosha and Dicer for processing [86]. This finding raises the debate on whether retroviruses can use the canonical miRNA pathway to produce miRNA again.

Besides miRNAs, retroviruses also encode lncRNAs to regulate viral or host genes expression [87]. In recent years, transposable elements (TEs) have been identified as a major lncRNA repertoire, and are treated as functional domains of lncRNAs due to their multiple RNA-, DNA-, and protein-binding properties [88]. Nearly two thirds of the mature lncRNAs are originated from transposable elements (TEs). Among them, ten percent are transcribed from endogenous retrovirus (ERV) [89]. HERV-H is one of the primate-specific endogenous retroviruses that is preferentially expressed in human embryonic stem cells (hESCs) and induced pluripotent stem cells (hiPSCs) [90, 91]. Nearly ten percent of the HERV-H transcripts are lncRNAs [92]. One of the HERV-H-driven lncRNAs was found to interact with the transcriptional coactivator p300 and pluripotency factor OCT4 and enriched in the HERV-H LTR7 regions, resulting in the activation of neighboring genes and in turn regulating pluripotency-related genes [93]. In addition to the regulation of hESCs, another ERV-derived lncRNA named Endogenous retroViral-associated ADenocarcinoma RNA (EVADR) was found to be expressed specifically in human adenocarcinoma but not in non-glandular origin tumors [94]. Human T cell leukemia virus type 1 (HTLV-1), a retrovirus of the human T-lymphotropic virus (HTLV) family, is the etiologic agent of adult T-cell leukemia (ATL) [95]. An HTLV-1-encoded protein named HTLV-1 bZIP factor (HBZ) was previously found to suppress the viral *Tax* expression [96]. Recently, researchers found that the mRNA of *HBZ* is retained in the nucleus and functions as an lncRNA to promote T cell proliferation [97, 98].

Taken together, some special features of retrovirus-derived miRNAs have emerged: (1) biogenesis—retroviruses use both canonical and non-canonical biogenesis pathways to generate miRNA; and (2) mode of action—retrovirus-encoded miRNA target can the promoter TATA box to upregulate gene transcription instead of targeting the mRNA 3′ UTR to suppress translation. These findings extend our understanding of the RNAi pathway in mammalian cells. Although the small size of retroviral RNA genomes has limited their coding ability for ncRNAs, the extraordinary diversity of viral transcripts and the enormous host gene regulation network enable the viral ncRNAs unlimited functional potentials.

### Cellular non-coding RNAs and retroviral gene expression

During productive infection, plenty of host factors are involved in the transcriptional regulation of viral genes such as the P-TEFb complex and NFκ-B [99, 100]. In addition, massive interactions between viral products and host proteins or ncRNAs occur at different levels. Therefore, viral gene expression might be the most regulated step in the retroviral life cycle.

#### Host non-coding RNAs regulate viral transcription and RNA transport

The efficient replication of HIV-1 is dependent on many cellular transcription factors, which could also be the targets of host RNAi. As a subunit of RNA polymerase II elongation factor P-TEFb, Cyclin T1 is required for Tat transactivation of HIV-1 LTR-directed gene expression [101]. MiR-198 is relatively highly expressed in human monocytes compared to macrophages, which are more permissive for HIV-1 replication. By targeting the 3′ UTR of Cyclin T1 mRNA and suppressing its translation, miR-198 functions to restrict HIV-1 replication in monocytes [102]. Many other host miRNA-mediated indirect effects on HIV-1 transcription through targeting the host dependency factors (HDFs) have been summarized by Barichievy et al. [103] (Fig. 1H).

Cellular lncRNAs also participate in the transcriptional regulation as well as the transportation of HIV-1 RNAs. A group identified six lncRNAs that were dysregulated by HIV-1 infection in both Jurkat and MT4 cells, one of which is NEAT1 (Nuclear Paraspeckle Assembly Transcripts 1) [104]. NEAT1 contributes to the formation of paraspeckles through sequestering multiple component proteins [105–107]. Some of the NEAT1-sequestered proteins are pivotal co-factors for efficient HIV-1 gene expression, including p54nrb, PSF, Matrin3, and RBM14 [108–110]. Besides canonical lncRNAs, some non-canonical lncRNAs were also involved in HIV-1 replication [46, 111, 112]. P-TEFb, which is composed of CDK9 and Cyclin T1, is indispensable for efficient HIV-1 transcription [113]. Two independent groups identified that lncRNA 7SK small nuclear RNA (snRNA) can negatively regulate HIV-1 Tat transcriptional activation by specifically sequestering P-TEFb [111, 112]. In addition, HIV-1 unspliced RNAs can be hijacked in NEAT1-supported nuclear paraspeckles and subjected to RNA editing. Knockdown of NEAT1 significantly enhanced HIV-1 production by increasing nucleus-to-cytoplasm export of Rev-dependent HIV-1 mRNAs containing an instability element (INS) [104].

#### Host non-coding RNAs directly target HIV-1 RNAs for post-transcriptional suppression

HIV-1 infection suppresses the RNA interfering pathway of host cells [114]. HIV-1 infection also causes global dysregulation of host miRNA expression profiles [115, 116]. HIV-1 Tat protein is shown to be a suppressor of RNA silencing (SRS), which abrogates the host cells RNA-silencing defense by subverting the ability of Dicer to process precursor double-stranded RNAs into siRNAs [117]. The HIV-1 TAR element was also reported to inhibit the host RNAi pathway by competitively binding to TAR RNA binding protein (TRBP), a cofactor of the key RNAi component Dicer [118]. These findings suggest that HIV-1 is escaping from the restriction imposed by the host RNAi mechanism. In fact, many host miRNAs have been shown to affect HIV-1 replication in direct or indirect ways.

Since HIV-1 is a RNA virus, its genomic RNA is a potential target for host miRNAs. Using in silico approaches, a number of miRNA binding sites in HIV-1 genomic RNA or mRNA transcripts were predicted and subsequently experimentally validated. Hariharan et al. reported that miR-29a and miR-29b were predicted to target HIV-1 Nef transcripts, whereas miR-149, miR-378, and miR-324-5p were predicted to target Vpr, Env, and Vif transcripts, respectively. However, these targeting sites were not located in the HIV-1 3′ LTR [119]. These authors then showed that the cellular miRNA miR-29a downregulates the expression of Nef protein and interferes with HIV-1 replication in HEK293T and Jurkat T cells [120, 121]. Similarly, Houzet et al. predicted the target sites of 22 human miRNAs in the HIV-1 genome, five of which were capable of inhibiting HIV-1 replication in 42CD4 cells derived from HEK293 cells stably expressing CD4 and CXCR4 [122]. Houzet et al. further demonstrated that the degree of complementarity between the predicted viral sequence and cellular miR-326 correlates with the potency of miRNA-mediated inhibition of viral replication. This finding indicates the selection pressure imposed on HIV-1 by the host RNAi pathway, which may drive the evolution of HIV-1. Using the photoactivatable ribonucleoside-induced cross-linking and immuno-precipitation (PAR-CLIP) technique, Whisnant et al. discovered several binding sites in the HIV-1 genome for cellular miRNAs, a subset of which were capable of repressing viral gene expression, including miR-423, miR-301a, and miR-155 [123]. They also argued that HIV-1 transcripts have evolved to avoid inhibition by host miRNAs by adopting extensive RNA secondary structures that occlude most potential miRNA binding sites.

Collectively, the current data show that cellular non-coding RNAs directly or indirectly modulate viral transcription, RNA transportation, and translation. Meanwhile, the viruses have been evolving to evade inhibition by the RNAi pathway, which is enabled by their hyper mutation rate as well as the complicated folding of viral RNAs.

### Non-coding RNAs and retrovirus assembly: the packaging of non-coding RNAs into retroviral particles

It was noticed several decades ago that host RNAs could be packaged into retroviral particles [124, 125]. Over 30% of the RNA in retroviral particles consist of host RNAs. These host RNAs include mRNA, tRNA, and small non-coding RNAs transcribed by Pol III. The most well-known host RNA packaged into HIV-1 virions is tRNA_Lys3_, which serves as the primer to initiate HIV-1 reverse transcription [126]. The non-coding RNA 7SL is another highly enriched host RNA identified in HIV-1 virions [127], which is important for the efficient packaging of APOBEC3G into virions [128]. Numerous subsets of endogenous retroelement RNAs expressed in virus-producing cells are also preferentially packaged. For example, intact endogenous retroviral transcripts like the murine VL30 elements are packaged by murine leukemia virus (MLV); and fragments of non-long terminal repeats (LTR) retroelements such as the transcripts of divergent and truncated Long INterspersed Elements (LINEs) are packaged into virions by HIV-1 [129, 130].

Through the NGS technology, a pile of host miRNAs was found to be selectively packaged into HIV-1 virions and affect viral infectivity (Fig. 1L). Using the SOLiD sequencing platform, Schopman et al. examined the miRNA profiling in a T cell line and several primary cell subsets before and after HIV-1 infection. They also examined the miRNAs in HIV-1 particles, and found that a small subset of the host miRNAs is dramatically concentrated in the virions by up to 115 folds [45]. Notably, three of the packaged miRNAs: miRNA-382, miRNA-223, and miRNA-150, are shown to contribute to HIV-1 latency [46]. Later, Bogerd et al. used the Illumina Hiseq 2000 system to investigate the packaging of cellular miRNAs into HIV-1 virions produced from CEM-SS T cells. However, they just found a 2- to 4-fold enrichment of five host miRNAs in virions. Among them, miR-155 and miR-92a were reported previously to weakly bind HIV-1 transcripts [123]. Interestingly, an artificial miRNA target site introduced into the viral genome resulted in 10- to 40-fold increase in the packaging of the cognate miRNAs into virions, which significantly inhibited HIV-1 virion infectivity [131]. Nevertheless, it remains to be clarified how these virion-enriched miRNAs exert their functions during the early phase of the HIV-1 life cycle. It is possible that these virion-enriched miRNAs can inhibit viral reverse transcription or regulate the genes needed for viral entry or uncoating, as we discussed above regarding the roles of ncRNAs in the early phase of retroviral life cycle.

## Non-coding RNAs and retroviral latency

Given the inhibitory effect on gene expression mediated by miRNAs, it is intriguing to investigate the roles of host miRNAs in HIV-1 latency. Our group reported that cellular miRNAs potently inhibit HIV-1 production in resting primary CD4^+^ T cells and contribute to HIV-1 latency [46]. We showed that the 3′ ends of HIV-1 messenger RNAs are targeted by several cellular miRNAs including miR-28, miR-125b, miR-150, miR-223, and miR-382, which are enriched in resting CD4^+^ T cells compared to activated CD4^+^ T cells. Specific inhibitors of these miRNAs activate HIV-1 protein translation in resting CD4^+^ T cells transfected with HIV-1 infectious clones as well as viral production from resting CD4^+^ T cells isolated from HIV-1-infected individuals on suppressive combined antiretroviral therapy (cART) [46]. This is the first report showing that cellular miRNAs contribute to HIV-1 latency, which adds a new layer to the mechanisms of the establishment of HIV-1 latency (Fig. 1M).

In addition to miRNAs, lncRNAs have also been found to be involved in the regulation of HIV-1 latency. Recently, we found a cellular lncRNA-noncoding repressor of NFAT (NRON), which is highly expressed in resting CD4^+^ T lymphocytes, contributes to HIV-1 latency [132]. Knockdown of NRON in latently infected resting CD4^+^ T cells significantly enhanced HIV-1 expression without activating the cells. Early studies showed that NRON acts as a negative regulator of transcription factor NFAT, which promotes HIV-1 expression by binding to the downstream of TAR [133, 134]. NRON hijacks NFAT in the cytoplasm to suppress NFAT-mediated viral gene activation. Another group also suggested that the NRON levels were reduced by the early viral accessory protein Nef and increased by the late protein Vpu [135]. However, we found that the expression of NFAT in HIV-1 latently-infected resting CD4^+^ T cells is quite low. The mutation of NFAT binding sites in the HIV-1 5′ LTR did not abolish NRON-mediated suppression of HIV-1 transcription. These findings indicate that the suppression of HIV-1 activation by NORN is independent of NFAT in resting CD4^+^ T cells. Intriguingly, the expression of the HIV-1 transactivator Tat decreased significantly upon NRON overexpression. Further investigations showed that NRON directly links Tat to the ubiquitin/proteasome components including CUL4B and PSMD11 to specifically induce Tat degradation, thus facilitating HIV-1 latency (Fig. 1N). Collectively, these data suggest that NRON modulates HIV-1 latency in both NFAT-dependent and -independent manners. In addition, another group reported that an HIV-1 antisense transcript called ASP interacts with polycomb repressor complex 2 (PRC2) and recruits PRC2 to the HIV-1 5′ LTR. This induces the accumulation of suppressive H3K27 trimethylation and reduces viral transcription, which facilitates the establishment of HIV-1 latency [136].

## Non-coding RNAs and retrovirus pathogenesis

Retrovirus infection causes severe diseases such as cancers and AIDS. As key regulators of cellular processes, non-coding RNAs are involved in the progression of diseases caused by retroviruses. We showed that HIV-1 infection decreases the expression of a cellular miRNA let-7i in CD4^+^ T cells by attenuating the transcription of its precursor [137]. Let-7i activates the transcription of cytokine interleukin-2 (IL-2) through targeting its promoter TATA box region [78]. It has been observed for a long time that HIV-1 infection causes a decrease in IL-2 levels, which is one of the causes of CD4^+^ T cell depletion, but the mechanism was not clear. Our findings reveal a novel pathway that the HIV-1 infection-induced suppression of the let-7i/IL-2 axis contributes to CD4^+^ T cell death [137]. Another interesting study showed that the BLV-miR-B4 shares an identical seed sequence with the cellular pro-oncogene miR-29, and both downregulate a similar set of mRNA targets [138]. Given that miR-29 overexpression is associated with B-cell neoplasms that resemble BLV-associated tumors, these findings suggest a possible miRNA-mediated mechanism contributing to BLV-induced tumorigenesis [66]. Other support for their roles in tumor onset and progression is that these miRNAs are highly expressed in preleukemic and malignant cells in which viral structural and regulatory gene expression was repressed [138]. The increasing ncRNA expression profiling data during viral infection are very helpful to identify those ncRNAs that are involved in retroviral pathogenesis.

## Application of non-coding RNA approaches in the specific treatment of retrovirus-related diseases

Since non-coding RNAs can sequence-specifically block retrovirus or host factors required for viral replication, non-coding RNAs are ideal candidates for the treatment of retrovirus infection. HIV-1 latency in resting CD4^+^ T cells is a major obstacle for the eradication of viruses from HIV-1-infected patients receiving combination antiretroviral therapy (cART) [3, 5]. The “shock and kill” strategy aims to activate the latently-infected viruses and induce the killing of the infected cells by specific cytotoxic effects [139]. Several approaches have been developed to activate latent virus transcription for killing, including by activating T lymphocytes with IL-2 or IL-2 plus anti-CD3/anti-CD28 antibody [140, 141], protein kinase C (PKC) activators (e.g. prostatin [142]), or activating transcription with histone deacetylases inhibitors (HDACi) without inducing host cell activation (such as valproic acid [VPA], suberoylanilide hydroxamic acid [SAHA]) [143–145]. However, the first approach has been shown to cause serious cytotoxic effects, while PKC agonists and HDACi are speculated to cause global gene expression activation with unpredictable side effects. Thus, an HIV-1 provirus-specific activating reagent is ideal for purging the latent reservoir. Our study demonstrated that the HIV-1-encoded miRNA miR-H3 could activate HIV-1 transcription in a sequence-specific manner [77] (Fig. 1K). Together with our previous findings that some cellular miRNAs contribute to the latency of HIV-1 by inhibiting HIV-1 production [46], a combination of the HIV-1 TATA box-targeting small RNA(s) and the inhibitors of these cellular miRNAs could provide an HIV-1-specific and much safer approach for activating and eradicating the HIV-1 latent reservoir [146]. In addition, lncRNAs could also be good candidates for activating HIV-1 latency. The depletion of NRON, especially in combination with a histone deacetylase (HDAC) inhibitor, significantly reactivates viral production from HIV-1-latently infected primary CD4^+^ T lymphocytes [132]. Collectively, our data demonstrate that non-coding RNAs could be used for the development of more effective and virus-specific latency-reversing agents [146]. Considering the significant sequence variations among different strains of retrovirus, such as HIV-1, even within a single infected individual [147], the efficacy of single small ncRNA, e.g. siRNA or miRNA, targeting the viral transcripts must vary as well. A combination of small ncRNAs that target multiple conservative sites of the viral sequence will prevent the rise of escape mutants. Moreover, the delivery efficiency of ncRNAs to infected cells needs to be further improved in the future.

## Conclusion and future perspectives

Recent advances in the study of ncRNAs have greatly improved our understanding of retroviral replication, pathogenesis, and evasion from the host immune surveillance. The advent of next-generation sequencing technology, in particular, has completely changed the field of non-coding RNA research. The NGS has two main advantages compared to the classic Sanger sequencing: (1) depth, the NGS is also called deep-sequencing due to its capability to capture extreme low profiling RNA transcripts; (2) accuracy, NGS can provide both quantitative expression data (reads) and the sequence of individual RNA molecules. The successful applications of NGS in retrovirus ncRNA research have been proven by the identification of retrovirus-derived small ncRNAs [129, 130] and host small RNAs packaged into viral particles [45]. Moreover, NGS-based novel technologies such as ChIP-Seq (Chromatin ImmunoPrecipitation-sequencing) and CLIP-Seq (CrossLinking ImmunoPrecipitation-sequencing) are powerful for functional analysis of non-coding RNAs [148, 149]. However, when NGS is used to study ncRNAs in a retrovirus system, some precautions need to be taken: (1) it is important to distinguish “noise” and “signal”, as many low- or even high-abundant RNAs are random degradation products instead of functional molecules. In such cases, functional assays must be conducted carefully with a variety of independent approaches [77]. (2) The usage of different cell types, different protocols of viral infection such as viral titer, infection time etc., will result in differing NGS data. Therefore, the comparison of NGS data from heterogeneous sample preparations is a reliable way to identify retrovirus-derived ncRNAs and infer their roles in viral infection [123].

The study of non-coding RNAs in the retrovirus system has revealed a whole new layer of viral replication and virus–host interactions. The reports cited in this review have shown that ncRNAs are involved in the key steps of the retroviral life cycle, especially during the late phase of infection: viral transcription, translation, and assembly, as well as latency and pathogenesis (Fig. 1, Tables 1, 2). In contrast, much less is known about the roles of ncRNAs in the early phase of the retrovirus life cycle, which deserve further investigation. The increasing understanding of ncRNAs and their interactions with retroviruses will eventually benefit the therapy of retrovirus-related diseases.
